# Isolation and Characterization of Chicken Serum Albumin (Hen Egg Alpha-Livetin, Gal d 5)

**DOI:** 10.3390/foods11111637

**Published:** 2022-06-01

**Authors:** Xingyi Jiang, Han Mu, Yun-Hwa Peggy Hsieh, Qinchun Rao

**Affiliations:** 1 Department of Nutrition and Integrative Physiology, Florida State University, Tallahassee, FL 32306, USA; xy1521@outlook.com (X.J.); yhsieh@fsu.edu (Y.-H.P.H.); 2 Novavax, Inc., Gaithersburg, MD 20878, USA; hamu@novavax.com

**Keywords:** chicken serum albumin, alpha-livetin, thermostability, extraction buffer, reducing agent, pH effect, linear epitope

## Abstract

Chicken serum albumin, i.e., hen egg alpha-livetin, is a recognized food allergen in chicken meat and hen eggs. Currently, there is no immunoassay available for its detection from food matrices. The characterization of chicken serum albumin-specific antibodies and the extraction of the target protein are essential for immunoassay development. One monoclonal antibody (mAb), 3H4, was used in this study due to its selectivity to a linear epitope on avian serum albumin. To study the extraction of chicken serum albumin, phosphate-buffered saline (PBS) with two additives, i.e., sodium dodecyl sulfate (SDS) and dithiothreitol (DTT), was used for its extraction from chicken blood plasma and hen egg yolk. SDS and DTT improved the chicken serum albumin’s recovery and enhanced chicken serum albumin’s immunodetection. In addition, chicken serum albumin retained the best solubility and immunoreactivity after heat treatment in a neutral condition. It experienced degradation and aggregation in acidic and alkaline conditions, respectively. Overall, PBS containing 0.1% SDS and 1 mM DTT (pH 7.2) was a better extraction buffer for chicken serum albumin. However, the complexity of the food matrix and elevated temperature could reduce its solubility and immunoreactivity.

## 1. Introduction

Eggs are one of the major allergenic foods [1], affecting approximately 0.9% of children [2] and 0.8% of adults [3] in the U.S. Besides the major allergens, such as ovomucoid (Gal d 1) and ovalbumin (Gal d 2) from egg white, alpha-livetin (Gal d 5) is the first identified hen egg yolk allergen [4]. Although alpha-livetin accounts for only around 1.9% (*w*/*w*) of hen egg yolk proteins [5], 100% of hen egg yolk allergic patients showed a reaction to it via in vitro immunoglobulin E (IgE) testing [6]. Alpha-livetin was also identified as an inhalant allergen causing bird-egg syndrome [7]. Hen egg alpha-livetin is identical to chicken serum albumin, which is transferred to the ovary and incorporated into the egg cell by receptor-mediated endocytosis [8]. Chicken serum albumin is commonly found in all tissues, such as blood and muscle tissues [9]. It occupies around 50% (*w*/*v*) of total chicken serum proteins [10]. Chicken serum albumin has been recognized as an allergen for chicken meat allergy [11]. The cross-reactivity between chicken and porcine serum albumin has been reported [12].

Chicken serum albumin is a 70 kDa protein with an isoelectric point (pI) of 5.51 [13]. It contains three domains with four to six α-helices in each domain [14] and is highly conserved in the three-dimensional structure [15]. Chicken serum albumin comprises 9 loops linked by 17 intramolecular disulfide bonds [13], contributing to protein stability and folding. Although chicken serum albumin shared around 40% similarity in amino acid sequence with mammalian serum albumin, their different properties were noticed. For example, chicken serum albumin was less likely to experience glycation compared to bovine serum albumin (BSA) [16].

As a cross-reactive allergen, it is necessary to investigate chicken serum albumin’s thermostability and immunodetection. Chicken serum albumin was a partially heat-labile allergen, indicating that heat (90 °C for 30 min) could not eliminate but could decrease its antigenicity in a hen egg yolk model by using immunoblot and enzyme-linked immunosorbent assay (ELISA) [11]. The IgE binding to chicken serum albumin in immunoblot was observed even after heating at 140 °C for 20 min [17]. As for the immunodetection of chicken serum albumin, the development of such immunoassays is dependent on the optimal extraction of the target protein from different food matrices and antibody selectivity. Chicken serum albumin is soluble in water, and its solubility is determined by various external factors, such as pH [18] and heat treatment [19]. The evaluation of the extraction buffer efficacy should be conducted based on at least two criteria. On the one hand, the recovery of the target analyte needs to be maximized. While on the other hand, the ingredients in the extraction buffer should not interfere with the antibody–antigen interaction and cause false detection results.

There are a few investigations on the effect of extraction buffer pH and composition on the thermostability and immunodetection of chicken serum albumin from different food matrices. One chicken serum albumin ELISA kit is commercially available, but it is applicable to analyze native serum albumin from biological samples [20]. Therefore, the objectives of this study were: (1) to characterize one anti-chicken serum albumin monoclonal antibody (mAb), (2) to isolate chicken serum albumin from chicken blood plasma, and (3) to investigate the effect of extraction buffer composition and pH on chicken serum albumin extractability, thermostability, and immunoreactivity.

## 2. Materials and Methods

### 2.1. Materials

Porcine and chicken whole blood were freshly collected from local farms (Tallahassee, FL, USA). During collection, one part of 3.8% (g/mL) sodium citrate was added to nine parts of blood. Chicken blood cells were obtained by the collection of precipitates after centrifuging chicken whole blood at 3000× *g* for 15 min at 4 °C. Chicken blood plasma was taken after the second centrifugation of the supernatant. Goat and sheep whole blood was purchased from LAMPIRE Biological Laboratories, Inc. (Pipersville, PA, USA). Bovine, horse, rabbit, and turkey whole blood was purchased from Hemostat Laboratories, Inc. (Dixon, CA, USA). All blood was stored at −20 °C until use.

Animal meats (beef shoulder steak, chicken thigh, pork loin, turkey breast, and Atlantic salmon fillet) and hen eggs were purchased from the local grocery store (Tallahassee, FL, USA). All meats were ground when received and stored at −80 °C until use. The hen egg yolk was carefully separated from the egg white. Chicken whole blood, chicken blood plasma, chicken blood cells, hen egg yolk, and hen egg white were lyophilized. Defatted hen egg yolk powder was prepared by adding 10 mL of chloroform to 1 g of lyophilized hen egg yolk powder, followed by filtration and vacuum evaporation. Commercial chicken serum albumin (CSA62) was purchased from Equitech-Bio, Inc. (Kerrville, TX, USA). All general chemicals and reagents were of analytical grade. All solutions were prepared using deionized water from a NANOpure DIamond ultrapure water system (Barnstead International, Dubuque, IA, USA).

### 2.2. Sample Preparation

Unless specified otherwise, (1) all the procedures were conducted at 4 °C, (2) heat treatment was performed at 100 °C for 15 min, 600 rpm, using a thermomixer (Eppendorf, Hamburg, Germany), (3) the sonication was performed at 50% amplitude for 10 s three times using a Q125 Sonicator (Qsonica, LLC., Newtown, CT, USA), (4) the homogenization was performed at 11,000 rpm twice using an ULTRA-TURRAX T-25 basic homogenizer (IKA Works, Inc., Wilmington, NC, USA), (5) all centrifugation was performed at 20,000× *g* for 15 min, and (6) filtration was performed using a Whatman No. 4 filter paper (Whatman, Maidstone, UK).

To study mAb selectivity and the target analyte using ELISA (Section 2.4) and immunoblot (Section 2.7), animal blood and meat protein extracts were prepared according to Jiang et al. [21], with modifications. Unheated blood liquid samples were 1:100 (*v*/*v*) diluted with 10 mM phosphate-buffered saline (PBS, pH 7.2), sonicated, and centrifuged to collect the supernatant. Four grams of unheated animal meat, heated animal meat, and heated animal blood were 1:1 (*w*/*w*) mixed with PBS. After homogenization, sonication, and 1 h rotation, the protein extracts were collected after being centrifuged twice and filtrated.

To study the extractability of chicken serum albumin from two powders, i.e., chicken blood plasma and hen egg yolk, four extraction buffers ((I) PBS, (II) PBS containing 0.1% (*w*/*v*) of sodium dodecyl sulfate (PBS-SDS), (III) PBS containing 1 mM dithiothreitol (PBS-DTT), and (IV) PBS-SDS containing 1 mM DTT (PBS-SDS-DTT)) were prepared. The pH of the four buffers was 7.2. Briefly, 2 mg of each powder was mixed with 2 mL of each extraction buffer. The mixture was rehydrated for 2 h at room temperature. After centrifuging twice, 1 g of the supernatant from each protein extract was heated. After centrifugation, the thermosoluble proteins were collected.

To study the effect of pH on chicken serum albumin, the pH of buffers I and IV was adjusted to 3 and 10. Two milligrams of commercial chicken serum albumin standards were mixed with buffers I and IV at three pH levels (pH 3, 7, and 10). After 1 h of rehydration at room temperature and centrifugation, 1 g of the supernatant from each protein extract was heated. After centrifugation, the thermosoluble proteins were collected.

To study the thermostability of chicken serum albumin, three models, i.e., commercial chicken serum albumin standard (Model 1), chicken blood plasma (Model 2), and hen egg yolk (Model 3), were established. Briefly, 4 mg of lyophilized commercial chicken serum albumin, chicken blood plasma, and hen egg yolk powder were mixed with 1 mL of buffers I and IV, respectively. After 1 h of rehydration at room temperature and centrifugation, 1 g of the supernatant from each protein extract was heated under four conditions (60 °C/15 min, 60 °C/30 min, 100 °C/15 min, and 100 °C/30 min). After centrifugation, the thermosoluble proteins were collected.

The protein concentration of all samples was determined using the BCA (bicinchoninic acid) assay, in which bovine serum albumin (BSA) was the protein standard (working range: 25–2000 µg/mL). The relative protein solubility (%) was calculated by the ratio of protein concentration of the heated one to the unheated one within the same model.

### 2.3. Antibody Development

Thermosoluble blood proteins from chicken and turkey whole blood (LAMPIRE Biological Laboratories, Pipersville, PA, USA) were used as poultry blood immunogens for monoclonal antibody (mAb) development, according to Jiang, Fuller, Hsieh, and Rao [21]. The animal immunization and antibody production were performed in the Hybridoma Facility at Florida State University (FSU, Tallahassee, FL, USA). All animal experiments were approved by the FSU Animal Care and Use Committee (ACUC).

mAbs were developed using the hybridoma technique [21]. Briefly, three female BALB/c mice (6–8 weeks old) were immunized subcutaneously and intraperitoneally with a total of 50 μg of the poultry blood immunogen mixed 1:1 (mL/mL) with Sigma Adjuvant System (SAS, Sigma-Aldrich, St. Louis, MO, USA), followed by 2 or 3 booster injections at 4-week intervals with 25 μg/mouse of the immunogen mixed 1:1 (mL/mL) with SAS. The supernatants were used for the initial screening against the immunogen using an indirect, non-competitive ELISA. For the secondary selection, positive cells from the initial screening were expanded to test for cross-reactivity with protein extracts from different animal species and plant sources. The cell lines selected were cloned twice by a limiting dilution method. Finally, one mAb, 3H4, was selected based on its desired affinity and selectivity. mAb3H4 was purified using a Bio-Scale Mini Affi-Prep Protein A Resin Cartridge (Bio-Rad Laboratories, Hercules, CA, USA). Its IgG isotype was determined using the mouse mAb isotyping reagents (Sigma-Aldrich) following the manufacturer’s instructions.

### 2.4. Indirect Non-Competitive Enzyme-Linked Immunosorbent Assay (ELISA)

ELISA was used to screen the selectivity of the developed mAbs according to Jiang, Fuller, Hsieh, and Rao [21], with modifications. The incubation of each step was at 37 °C for 1 h, and between each step, the microplate was washed with PBST (PBS containing 0.05% (*v*/*v*) Tween 20) three times. Briefly, 100 ng/well of samples (whole blood, meat, and egg extracts) diluted in 50 mM carbonate-bicarbonate buffer (pH 9.6) was coated onto a 96-well polystyrene high-binding microplate (Corning Inc., Corning, NY, USA). After coating, each well was blocked with 200 μL of 1% (*w*/*v*) BSA in PBS (BSA-PBS). Then, mAb3H4 diluted in the antibody buffer (BSA-PBST, 1% (*w*/*v*) BSA in PBST) was added. After the primary antibody incubation, 2.87 ppm of goat anti-mouse IgG (Fc specific) horseradish peroxidase (HRP) conjugate (anti-IgG-HRP, A2554, RRID: AB_258008, Sigma-Aldrich) in BSA-PBST was added. After incubation and five washes, the color was developed using ABTS (2,2’-azino-bis(3-ethylbenzothiazoline-6-sulfonic acid)) substrate and its optical density was measured at 415 nm.

### 2.5. Isolation of Chicken Serum Albumin

Chicken serum albumin was isolated from chicken blood plasma using ammonium sulfate (AMS) precipitation followed by anion exchange chromatography according to Odunuga and Shazhko [22], with modifications. Unless specified otherwise, all subsequent procedures were conducted at 4 °C, and all centrifugation was performed at 10,000× *g* for 15 min. Briefly, 5 g of liquid chicken blood plasma was taken to 60% AMS saturation. After 2 h of rotation and centrifugation, the supernatant was further taken to 70% AMS saturation and rotated overnight. After centrifugation, the precipitates were suspended in 5 mL of the elution buffer (20 mM Tris-HCl containing 1 M NaCl, pH 9), mixed with four parts (*v*/*v*) of acetone, and incubated at −20 °C for 1 h. Two parts (*v*/*v*) of acetone were used to wash the precipitates collected after the centrifugation (15,000× *g* for 15 min). The precipitates after the second washing were air-dried and resuspended in 5 mL of the binding buffer (20 mM Tris-HCl, pH 9), followed by 1 h of rehydration at room temperature.

Anion exchange chromatography was performed using the Bio-Rad Econo System. Unless specified otherwise, (1) the flow rate was set as 1 mL/min, and (2) the sample loading, elution, and buffer equilibration were monitored using an EM-1 Econo UV (280 nm) Monitor. Briefly, the supernatant collected after centrifugation and filtration through a 0.45 µm syringe filter was loaded to a binding buffer pre-equilibrated Bio-Scale Mini UNOsphere Q Cartridge (Bio-Rad) at a speed of 0.5 mL/min. The sample was reloaded another two times to maximize protein binding. The Q Cartridge was washed with the binding buffer until no protein absorption was observed at 280 nm. Protein elution was carried out by a 17 mM stepwise increment of NaCl concentration. The absorbance of each fraction (1 mL) was measured at 280 nm.

### 2.6. Gel Electrophoresis

Sodium dodecyl sulfate-polyacrylamide gel electrophoresis (SDS-PAGE) was performed to study: (1) the protein profile from meat, blood, and egg extracts under reducing conditions, (2) the protein profile during chicken serum albumin isolation under reducing conditions, (3) the effect of extraction buffer (composition and pH) on the extractability and molecular integrity of chicken serum albumin under non-reducing and reducing conditions, and (4) the effect of heat treatment on the protein profile of chicken serum albumin, chicken blood plasma, and hen egg yolk under reducing conditions according to Jiang, Fuller, Hsieh, and Rao [21], with modifications. Briefly, various protein samples were mixed with 2× Laemmli sample buffer with or without 5% (*v*/*v*) β-mercaptoethanol and separated on SDS-PAGE (4% stacking gel and 15% separating gel), respectively. The protein profile was visualized using Coomassie blue staining.

### 2.7. Western Blot

Western blot was performed to study (1) mAb3H4 selectivity, (2) the effect of extraction buffer (composition and pH) on chicken serum albumin immunoreactivity, and (3) the effect of heat treatment on chicken serum albumin immunoreactivity. The separated protein bands from SDS-PAGE were transferred to a 0.45 µm nitrocellulose membrane (Bio-Rad). After verification using Ponceau S staining, the membrane was blocked using BSA-PBST for 1 h at room temperature. The membrane was further incubated with 1 ppm of mAb3H4 overnight at 4 °C, followed by 172 ppb of anti-IgG-HRP for 1 h at room temperature. The membrane was washed with PBST several times between each step. All images from non-reducing and reducing SDS-PAGE and Western blot were captured by the Azure c600 Imaging System and analyzed using the AzureSpot software (version 2.0.062, Azure Biosystems Inc., Dublin, CA, USA). For the same model, the relative immunoreactivity of chicken serum albumin was calculated based on the chicken serum albumin band intensity in heated samples compared with their unheated ones.

### 2.8. Statistical Analysis

The comparison of relative protein solubility and immunoreactivity within the same model among different heating conditions was performed using two-way analysis of variance (ANOVA) with Tukey’s post-test (GraphPad Prism, version 9.0.0 for Windows, GraphPad Software, Inc., San Diego, CA, USA). All experiments were independently repeated at least twice. *p* < 0.05 was considered statistically significant.

## 3. Results and Discussion

### 3.1. Antibody Characterization

#### 3.1.1. Antibody Target Analyte

**Figure 1:**
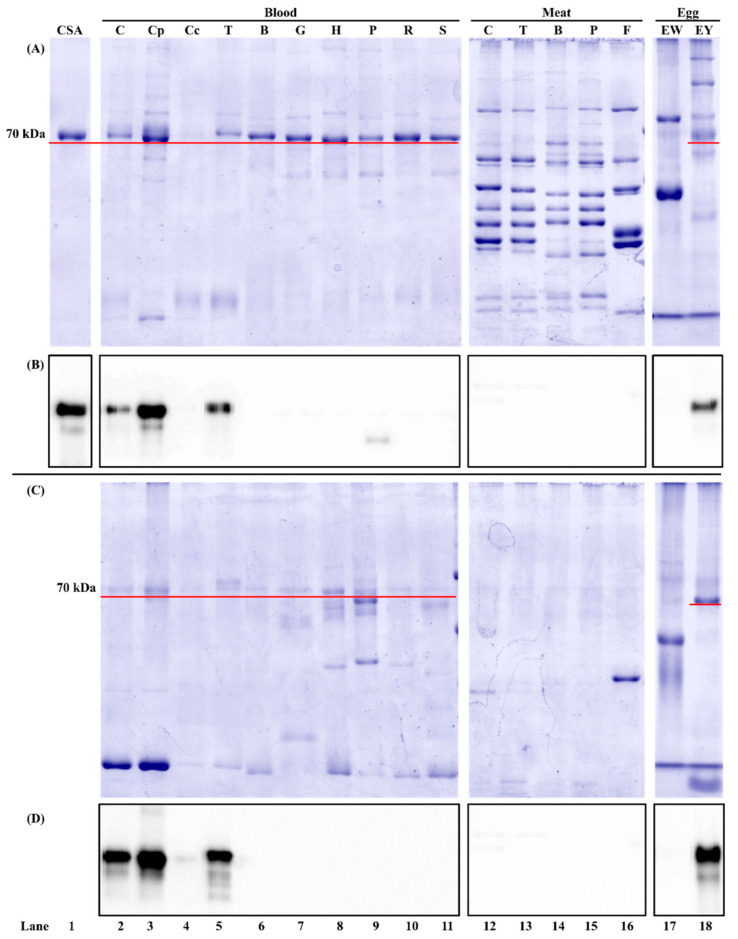
Reducing SDS-PAGE and its corresponding immunoblot probed with mAb3H4 using unheated (**A**,**B**) and heated (**C**,**D**) blood, meat, and egg samples. The protein loading mass was 1.5 µg/lane for blood, meat, and egg samples and 0.5 µg/lane for commercial chicken serum albumin standard (CSA). The IgG concentration of mAb3H4 was 0.75 ppm. C: chicken; Cp: chicken blood plasma; Cc: chicken blood cells; T: turkey; B: bovine; G: goat; H: horse; P: porcine; R: rabbit; S: Sheep; F: Atlantic salmon; EW: hen egg white; EY: hen egg yolk. The 70 kDa protein was indicated using a solid red line.

The in-gel protein profile of animal meat, blood, and hen egg is shown in Figure 1. As a control, commercial chicken serum albumin showed a 70 kDa band on the stained gel (Figure 1A, lane 1). For the unheated protein extracts, one major protein band at around 70 kDa was observed in all whole blood (Figure 1A, lanes 2, 5–11). This protein was a blood plasma protein because it existed in chicken blood plasma (Figure 1A, lane 3) instead of chicken blood cells (Figure 1A, lane 4). This protein band was also observed in hen egg yolk (Figure 1A, lane 18) but not in egg white (Figure 1A, lane 17). For the heated protein extracts, the protein profile from each sample was different from that of its unheated counterpart (Figure 1A,C). Fewer protein bands were observed in the heated sample due to the removal of non-thermostable proteins. For example, some high molecular weight proteins in animal meat disappeared after heat treatment (Figure 1A,C, lanes 12–16). It should be noted that the 70 kDa band was still visible in animal whole blood, chicken blood plasma, and hen egg yolk (Figure 1C, solid red line, lanes 2, 3, 5–11, 18). From Western blot, the target analyte of mAb3H4 is chicken serum albumin, as evidenced by the immunoreactive band in chicken serum albumin standard (Figure 1B, lane 1). The positive protein band was also observed in chicken whole blood, chicken blood plasma, and hen egg yolk, regardless of heat treatment (Figure 1B,D, lanes 2, 3, 5, and 18), confirming that the 70 kDa protein is chicken serum albumin. The antigenic band below 70 kDa (Figure 1B,D, lanes 2, 5, and 18) might be the degraded peptide(s) from serum albumin during sample preparation.

#### 3.1.2. Antibody Species Selectivity

mAb3H4 could react with serum albumin from chicken and turkey without cross-reaction with non-poultry species (Figure 1B,D). This confirmed the ELISA results (Table 1) showing that the positive immunosignal was observed in poultry blood and hen egg yolk. Although a weak protein band at around 50 kDa was observed in the unheated porcine blood (Figure 1B, lane 9) and a positive immunosignal was noticed in ELISA (Table 1), it was caused by the cross-reaction between porcine IgG and the secondary antibody (i.e., anti-IgG-HRP). It was reported previously that due to the IgG similarity among species, anti-IgG-HRP could falsely immunodetect porcine IgGs, leading to the false-positive immunosignal [23].

Chicken serum albumin, also known as α-livetin, is the most abundant plasma protein, which is essential for providing an osmotic effect and helping nutrient and hormone transportation [25]. Avian serum albumin showed a high resemblance among species. The amino acid similarity between chicken and turkey serum albumin was 91% [15], which explained this mAb’s reactivity to both chicken and turkey. Plasma serum albumin concentration varies depending on the physiological state, and around 15–20 g/L of plasma serum albumin was reported in chicken [26]. Besides chicken blood plasma, this protein can be found in muscle tissues and hen egg yolk [9]. Hen egg is reported to contain around 0.6% (*w*/*w*) of livetin [27,28]. Serum albumin from both muscle tissues and hen egg yolk was recognized as a food allergen, named Gal d 5. Patients with a hen egg yolk allergy showed IgE binding to Gal d 5 in ELISA and Western blot [11]. Currently, the IgE epitope(s) of chicken serum albumin has never been reported. In BSA, amino acid 524–542 has been considered a critical epitopic area for human allergy [29].

### 3.2. Chicken Serum Albumin Isolation

**Figure 2:**
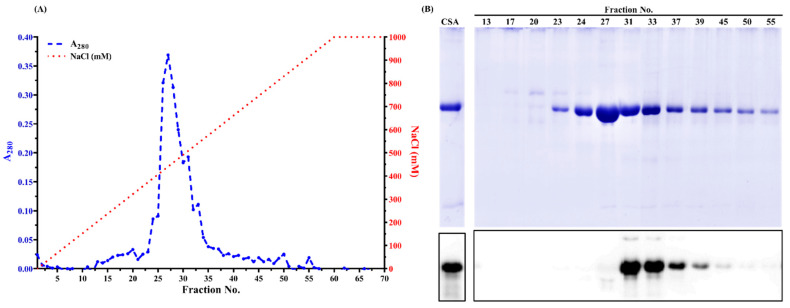
(**A**) Linear gradient protein elution profiles of anion exchange chromatography. The blue dashed line is the UV spectra of eluted fractions at 280 nm. The red dotted line is the linear NaCl gradient (17 mM/fraction). (**B**) Reducing SDS-PAGE of selected fractions and its corresponding immunoblot probed with mAb3H4. The protein loading mass was 1 µg/lane for commercial chicken serum albumin standard (CSA). Each fraction from anion exchange chromatography was 1:1 (mL/mL) mixed with 2× Laemmli buffer containing 5% β-mercaptoethanol. The loading volume was 13 µL/lane. The IgG concentration of mAb3H4 was 0.75 ppm.

During AMS precipitation, different protein profiles were observed. The 70 kDa protein band was more prominent as a function of AMS concentration (Figure S1). Compared to the protein profile in chicken blood plasma, many proteins at around 25 kDa and above 75 kDa were partially removed after 70% AMS precipitation (Figure S1, lanes 2–8). During anion exchange chromatography, one major peak was observed in fractions No. 24 to No. 36 (Figure 2A). From SDS-PAGE (Figure 2B), some impure proteins were removed when the NaCl concentration was less than 200 mM. A 70 kDa protein was visible from fractions No. 23 to No. 55 (Figure 2B). Interestingly, from Western blot (Figure 2B), chicken serum albumin was only detected in fractions No. 31 to No. 55, suggesting that the 70 kDa protein from fractions No. 23 to No. 31 is not serum albumin. Instead, it is chicken IgY, which was verified by Western blot using an anti-chicken IgY antibody. From Figure S2 (lanes 2 and 9), intense IgY bands were observed under both reducing and non-reducing conditions from chicken blood plasma. A similar immunopattern was also observed in the collected fractions (Figure S2, lanes 3–7 and 10–14), while it was not visible in pure chicken serum albumin standard (Figure S2, lanes 1 and 8) by using an anti-IgY antibody. Chicken IgY (180 kDa) is abundant in blood plasma and hen egg yolk and can be purified using anion exchange chromatography [30]. Under the reducing condition, it is dissociated into two heavy chains (~70 kDa) and two light chains (~25 kDa) [31], in which the heavy chain showed an overlap with the chicken serum albumin. It suggested that column chromatography could not completely separate livetin from IgY, which was also reported by Wang et al. [32].

### 3.3. Effect of Extraction Buffer Composition on Chicken Serum Albumin Extractability and Immunoreactivity

**Figure 3:**
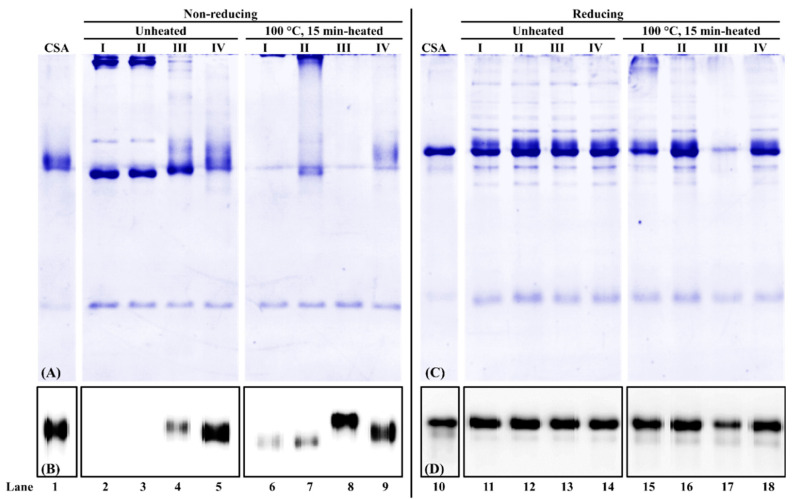
PAGE of chicken serum albumin (CSA) extractability and immunoreactivity from chicken blood plasma and its corresponding immunoblot under non-reducing (**A**,**B**) and reducing (**C**,**D**) conditions. (I) PBS, (II) PBS-SDS, (III) PBS-DTT, and (IV) PBS-SDS-DTT. The protein loading mass was 1 µg/lane for commercial chicken serum albumin standard (CSA) and 1.5 µg/lane for unheated/heated chicken blood plasma proteins. The IgG concentration of mAb3H4 was 0.75 ppm.

For chicken serum albumin extraction from chicken blood plasma powder, under the non-reducing condition, first, the total buffer-soluble protein profile was different among the four buffers. In the absence of a reducing agent (DTT) in the extraction buffer, high molecular weight proteins were observed in unheated and heated samples (Figure 3A, lanes 2–3 and 6, 7). The smearing protein bands at around 70 kDa were observed at all four extraction buffers (Figure 3A, lanes 2–9), with the intensity stronger in the unheated sample than that from the heated sample. Second, for unheated protein extracts, no immunoreactive chicken serum albumin was observed from PBS and PBS-SDS extraction (Figure 3B, lanes 2, 3). For heated protein extracts, chicken serum albumin was immunodetected in all four extraction buffers (Figure 3B, lanes 6–9). When DTT was added to the extraction buffer, the chicken serum albumin immunoreactivity increased 78% and 66% compared to PBS and PBS-SDS, respectively (Figure 3B, lanes 6–8). It suggested that mAb3H4 was specific to a linear epitope that was exposed in the presence of a reducing agent.

Under the reducing condition, from SDS-PAGE (Figure 3C, lanes 11–18), more protein bands at various molecular weights were observed. When β-mercaptoethanol was added, high molecular weight proteins decreased (Figure 3A,C, lanes 2–3 and 11–12). Regardless of heat treatment and extraction buffer composition, their band patterns were similar (Figure 3C, lanes 11–16, 18), except that the total lane intensity from PBS-DTT extraction was the weakest (Figure 3C, lane 17). In addition, no significant difference in chicken serum albumin immunoreactivity was observed among different extraction buffers (Figure 3D, lanes 11–14).

**Figure 4:**
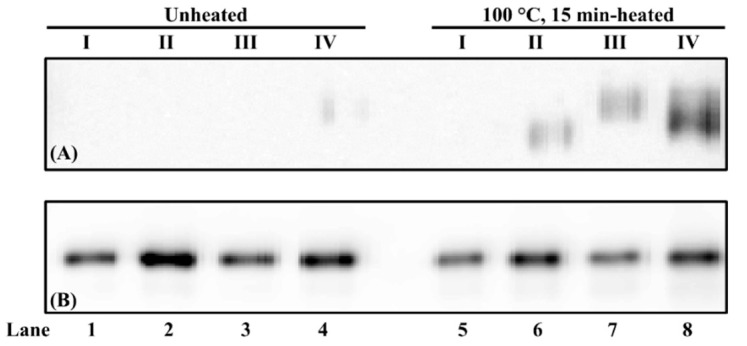
Immunoblot of chicken serum albumin (CSA) from hen egg yolk under non-reducing (**A**) and reducing (**B**) conditions. (I) PBS, (II) PBS-SDS, (III) PBS-DTT, and (IV) PBS-SDS-DTT. The protein loading mass was 1.5 µg/lane. The IgG concentration of mAb3H4 was 0.75 ppm.

As for chicken serum albumin extraction from defatted hen egg yolk powder (Figure 4), similar phenomena were observed in the chicken blood plasma matrix (Figure 3). First, under the non-reducing condition, weak chicken serum albumin in unheated hen egg yolk was immunodetected from PBS-SDS-DTT extraction (Figure 4A, lanes 1–4). For heated hen egg yolk, a hierarchy for immunodetectable chicken serum albumin is PBS-SDS-DTT > PBS-DTT > PBS-SDS > PBS (Figure 4A, lanes 5–8). Second, immunoreactive chicken serum albumin was observed in all samples under the reducing condition (Figure 4B).

A reducing agent such as DTT not only increased chicken serum albumin extractability but also enhanced its immunoreactivity. DTT has a redox potential of −0.33 V, which can reduce disulfide bonds in proteins and change protein conformation [33]. Chicken serum albumin contains 35 cysteine residues and can form up to 17 intramolecular disulfide bonds [13], leading to a unique secondary structure of double loops [34]. The remaining unpaired cysteine can interact with other redox-active proteins. For example, it is responsible for albumin dimerization by forming an intermolecular disulfide bond [35]. DTT was found to be a crucial component during chicken serum albumin extraction. It is reported that a reducing agent retained the extraction of BSA from heat- and storage-induced SS-linked serum albumin aggregates [36]. DTT could also change the serum albumin’s immunoreactivity. Human IgE immunoblots have shown that the immunoreactivity with BSA decreased after the reduction of disulfide bonds [37,38,39], while our studies showed that DTT could improve the immunoreactivity. It is possible that the mAb3H4 has a linear epitope which was exposed after the break of intramolecular disulfide bonds.

Both SDS and heating were noticed to affect chicken serum albumin’s immunodetection. For both blood plasma and hen egg yolk, the addition of SDS improved the chicken serum albumin’s immunosignal due to the following two reasons. First, SDS could disrupt non-covalent interactions of proteins [40], releasing serum albumin from the protein complex. Second, SDS could also bind to serum albumin and lead to its conformational change [41]. Additionally, heat-induced protein denaturation [42,43] and protein conformational change [39] could affect serum albumin’s immunoreactivity.

### 3.4. Effect of Extraction Buffer pH on Chicken Serum Albumin Solubility and Immunoreactivity

**Figure 5:**
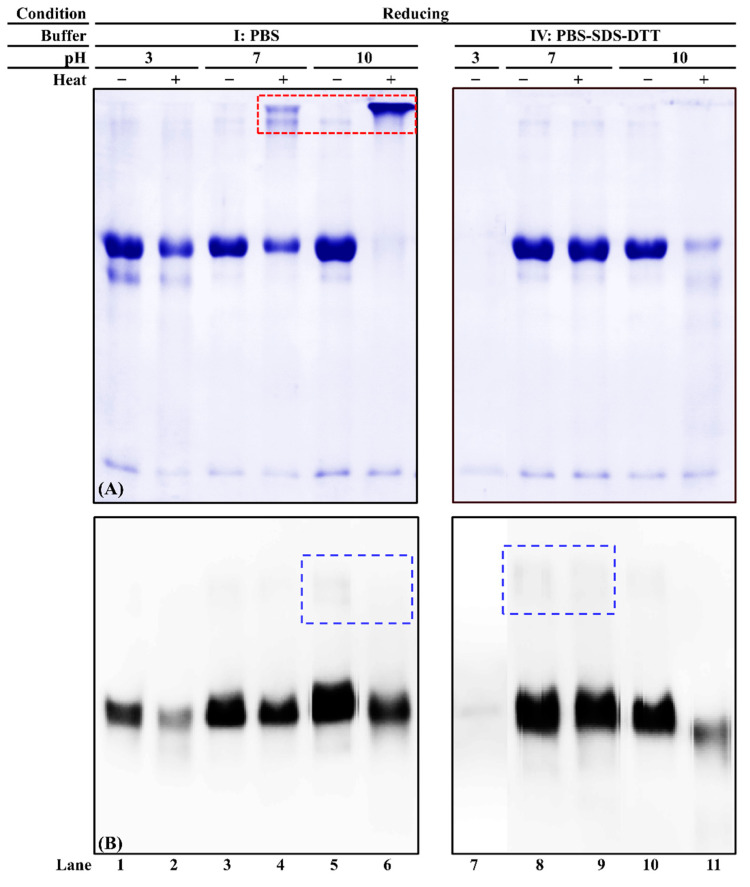
PAGE of chicken serum albumin (CSA) extractability and immunoreactivity from chicken blood plasma (**A**) and its corresponding immunoblot (**B**) under reducing conditions. “−”/“+”: Samples were unheated or heated at 100 °C for 15 min. The loading volume for each sample was 13 µL. The IgG concentration of mAb3H4 was 0.75 ppm. Chicken serum albumin aggregates and dimers were indicated using a red dotted box and a blue dashed box, respectively.

Three pH levels (acidic, neutral, and alkaline) of two buffers (PBS and PBS-SDS-DTT) were used to evaluate commercial chicken serum albumin’s solubility and immunoreactivity. When PBS was the solvent, an intense chicken serum albumin band was observed in unheated samples at all three pH levels (Figure 5A, lanes 1, 3, and 5). While in the heated protein extracts, a smearing band was observed at pH 10 (Figure 5A, lanes 2, 4, and 6). Thermosoluble chicken serum albumin aggregates were observed at pH 7 and 10 (Figure 5A, lanes 4 and 6). When PBS-SDS-DTT was used, for unheated samples, the chicken serum albumin band intensity was the weakest at pH 3 (Figure 5A, lanes 7, 8 and 10). Its solubility decreased at pH 10 after heat treatment (Figure 5A, lanes 10, 11). Compared to PBS, fewer chicken serum albumin aggregates were visible after heat treatment (Figure 5A, lanes 4, 6, 8, and 10).

The immunoreactivity of heated chicken serum albumin decreased compared to the unheated ones at each pH level of PBS extraction (Figure 5B, lanes 1–6). A weak dimer could be observed (Figure 5B, blue dashed box), suggesting that the disulfide bond was not completely dissociated. However, the immunoreactive aggregates were not observed. When chicken serum albumin was extracted using PBS-SDS-DTT, the weakest immunoreactivity was observed at acidic pH (Figure 5B, lane 7). The best extraction buffer was PBS-SDS-DTT (pH 7) in that the best extractability and immunoreactivity were preserved regardless of heat treatment.

It is known that pH affects protein solubility and conformation. Few studies were performed on the effect of pH on chicken serum albumin. For serum albumin from other species, Estey et al. [44] reported degradation of BSA under the acidic condition. The BSA molecules stabilized due to the decrease of hydrogen bonding as a function of pH increment [45]. At neutral and alkaline pH levels, the serum albumin underwent pH-induced conformation change [46], which led to different immunoreactivity. In addition, this study showed that at acidic pH, even in the presence of DTT, the solubility of chicken serum albumin was the weakest. This is because DTT is not functional when pH < 7 [47]. The optimal pH range for DTT is between 7 and 8 [48].

### 3.5. Effect of Buffer Composition on Chicken Serum Albumin Thermostability from Different Food Matrices

Relative protein solubility results showed that PBS-SDS-DTT retained more soluble chicken serum albumin than PBS (Table 2). Within each model, the soluble protein concentration decreased significantly when PBS was used (*p* < 0.05). After 100 °C/30 min heat treatment, the soluble proteins in Model 1 (chicken serum albumin standard), Model 2 (chicken blood plasma), and Model 3 (hen egg yolk) decreased by 21%, 26%, and 26%, respectively. While for PBS-SDS-DTT extraction, no significant difference in protein concentration was observed among each heat treatment.

**Figure 6:**
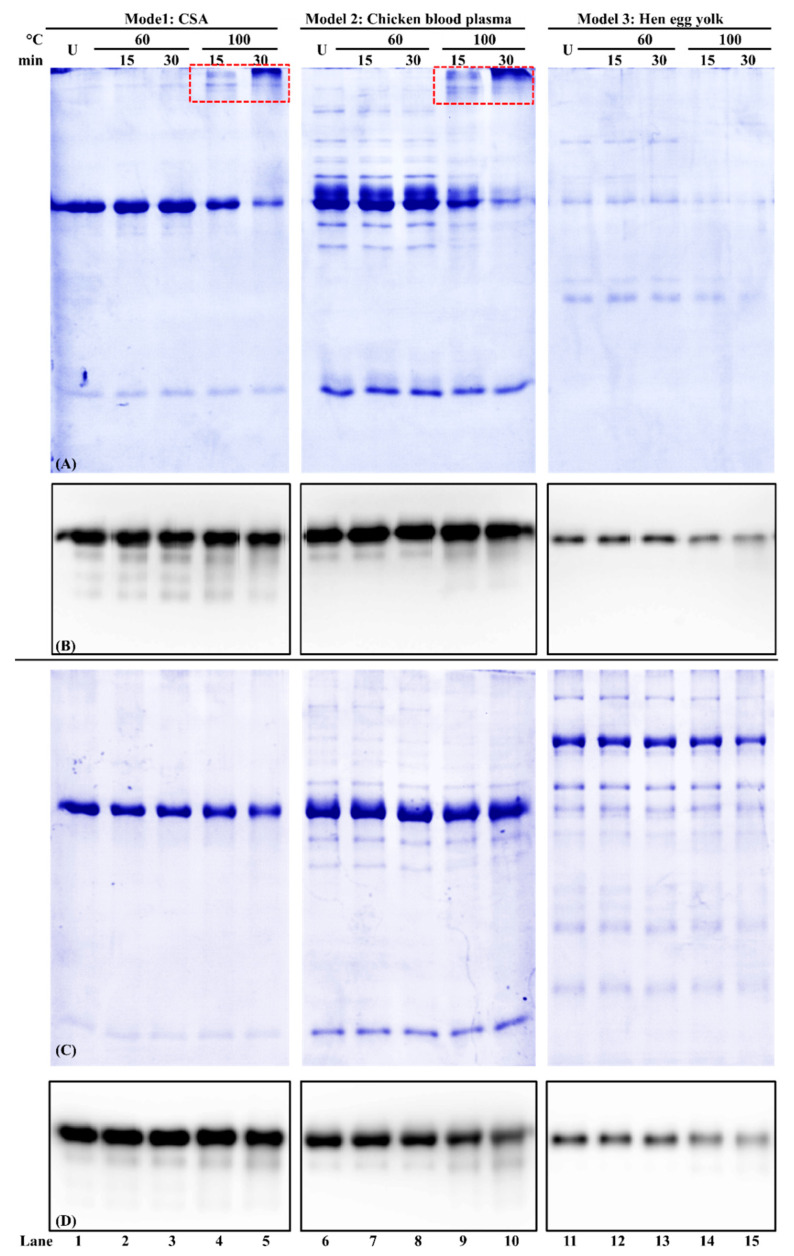
Reducing SDS-PAGE of chicken serum albumin (CSA) thermostability from three models and its corresponding immunoblot using PBS (**A**,**B**) and PBS-SDS-DTT (**C**,**D**) as the extraction. U: unheated. Sample loading volume: 13 µL/lane. The IgG concentration of mAb3H4 was 0.75 ppm. Chicken serum albumin aggregates were indicated using the red dotted box.

The protein profile of these samples was shown in reducing SDS-PAGE (Figure 6A,B). In Model 1 of the commercial chicken serum albumin standard, some high molecular weight chicken serum albumin aggregates were observed when PBS was used as a buffer (Figure 6A, lanes 1–5). When the heating condition was elevated to 100 °C for 30 min, the chicken serum albumin at the 70 kDa band intensity decreased (Figure 6A,B, lane 5), which was consistent with the protein relative solubility results (Table 2). Such a decrease was also observed in Models 2 and 3 (Figure 6A, lanes 6–15). For PBS-SDS-DTT extraction, there was no difference between each treatment within each model (Figure 6C).

For the immunoreactivity analysis, for both PBS and PBS-SDS-DTT extraction, no significant difference was observed in Model 1 between unheated and 100 °C/30 min heated chicken serum albumin (Figure 6B,D, lanes 1–5 and Table 2). For PBS-SDS-DTT extraction, although chicken serum albumin immunoreactivity was not changed in Model 2, it decreased significantly (*p* < 0.05) in Model 3 of hen egg yolk (Figure 6B,D, lanes 11–15 and Table 2).

In this study, thermostability is defined as the ability of chicken serum albumin to retain its solubility, molecular integrity, and immunoreactivity after heat treatment. First, for solubility, both buffer composition and food matrix affect the solubility of chicken serum albumin. In the chicken blood plasma and hen egg yolk, due to the presence of many other proteins, heat-induced protein precipitation was observed, leading to a decrease in the soluble protein concentration. Second, for molecular integrity, chicken serum albumin soluble aggregates were observed when the heating temperature reached 100 °C. At 80 °C, BSA experienced the initial stage of aggregation by the formation of aggregates [49] through hydrophobic and electrostatic interactions [50]. Third, for immunoreactivity, heat treatment was reported to affect the IgE binding to chicken serum albumin. For example, an 88% reduction of IgE reactivity after heating chicken serum albumin at 90 °C for 30 min [11]. Similar findings were also noticed in BSA, which lost its antigenicity after heating at 75 °C for 20 min [51].

The difference in chicken serum albumin’s antigenicity from different models was caused by the food matrix. For the commercial chicken serum albumin standard (Model 1), the sample matrix was simple. The thermal denaturation temperature of livetins in water was between 80 and 85 °C [5]. Our study showed that chicken serum albumin could still be immunodetected after denaturation. For both Model 2 and Model 3, the sample matrix was more complicated. Besides chicken serum albumin, other blood plasmas and yolk proteins were present, leading to protein–protein interactions. The extractability of chicken serum albumin from these two models depended on the heating conditions and the extraction buffer. Many studies have reported a decrease in protein recovery for thermally processed foods. For example, the protein recovery significantly reduced for the egg after heat treatment [51]. The addition of ionic detergent and the reducing agent was able to break the covalent and non-covalent interactions between proteins, thus improving the protein recovery. For example, 4% SDS achieved the best extraction of egg proteins out of the egg matrix [52].

## 4. Conclusions

Chicken serum albumin was isolated from the chicken blood plasma. One anti-chicken serum albumin mAb was characterized in this study to demonstrate the effect of extraction buffer on chicken serum albumin’s extractability, solubility, and thermostability. First, the addition of reducing agents such as DTT during the extraction process could enhance extractability by breaking inter- and intra-molecular disulfide bonds. Compared to the non-reduced samples, the solubility and immunoreactivity of chicken serum albumin increased significantly. Second, both acidic and alkaline pH levels were not favored during the extraction. They decreased the solubility of chicken serum albumin, especially from the heated samples. Third, chicken serum albumin from two food matrices (i.e., chicken blood plasma and hen egg yolk) was immunodetected after heating. Heating at 60 °C did not alter the immunoreactivity, while heating at 100 °C significantly decreased the immunoreactivity. Overall, PBS-SDS-DTT (pH 7.2) was a better extraction buffer than the other buffers studied. It not only increased the total protein recovery but also improved chicken serum albumin’s immunodetection.

## Figures and Tables

**Table 1:** Species selectivity of mAb3H4 using indirect, non-competitive ELISA.

	Reaction Unheated	Heated (100 °C, 15 min)
Animal whole blood		
Bovine	−	−
Goat	−	−
Horse	−	−
Porcine	+ ^a^	−
Rabbit	−	−
Chicken	+	++
Turkey	−	++
Animal meat		
Bovine	−	−
Pork	−	−
Salmon	−	−
Chicken	−	−
Turkey	−	−
Hen egg		
Egg white	−	−
Egg yolk	+	+++

+++, Very strong reaction (A_415_ > 1.2); ++, strong reaction (1.2 > A_415_ > 0.7); +, weak reaction (0.7 > A_415_ > 0.2); −, no reaction (A_415_ < 0.2) [24]. ^a^ The positive immunosignal in porcine whole blood was caused by the cross-reaction between porcine IgGs and secondary antibody.

**Table 2:** Comparison of relative protein solubility and relative immunoreactivity of chicken serum albumin from three models as a function of heat treatment.

Extraction Buffer	I: PBS (Mean ± SEM *, %)			IV: PBS-SDS-DTT (Mean ± SEM *, %)		
Sample Model	Model 1: Chicken Serum Albumin	Model 2: Chicken Blood Plasma	Model 3: Hen Egg Yolk	Model 1: Chicken Serum Albumin	Model 2: Chicken Blood Plasma	Model 3: Hen Egg Yolk
*Relative protein* *solubility*						
Unheated	100 ± 0 ^a^	100 ± 0 ^a^	100 ± 0 ^a^	100 ± 0 ^a^	100 ± 0 ^a^	100 ± 0 ^a^
60 °C/15 min	98.3 ± 0.5 ^a^	94.2 ± 0.9 ^b^	91.8 ± 1.6 ^b^	93.6 ± 0.5 ^a^	99.3 ± 1.9 ^a^	90.5 ± 3.9 ^a^
60 °C/30 min	97.4 ± 1.9 ^a^	90.9 ± 0.9 ^b^	92.2 ± 1.0 ^b^	88.6 ± 4.4 ^a^	99.0 ± 1.0 ^a^	97.5 ± 0.8 ^a^
100 °C/15 min	84.8 ± 0.3 ^b^	77.7 ± 2.3 ^c^	78.1 ± 0.7 ^c^	85.1 ± 5.6 ^a^	95.4 ± 7.2 ^a^	89.3 ± 4.6 ^a^
100 °C/15 min	79.8 ± 0.7 ^c^	73.9 ± 1.1 ^c^	74.1 ± 0.9 ^c^	92.7 ± 8.9 ^a^	93.5 ± 2.9 ^a^	95.6 ± 10.1 ^a^
*Relative* *immunoreactivity*						
Unheated	100 ± 0 ^a^	100 ± 0 ^a^	100 ± 0 ^a^	100 ± 0 ^a^	100 ± 0 ^a^	100 ± 0 ^a^
60 °C/15 min	94.1 ± 2.7 ^a^	115.7 ± 2.2 ^b^	91.0 ± 3.4 ^ab^	111.7 ± 3.5 ^ab^	103.3 ± 4.0 ^a^	86.5 ± 1.8 ^ac^
60 °C/30 min	101.8 ± 2.4 ^ac^	111.7 ± 1.9 ^b^	88.0 ± 3.0 ^b^	111.9 ± 6.7 ^ab^	103.1 ± 1.8 ^a^	84.2 ± 1.5 ^b^
100 °C/15 min	110.7 ± 2.7 ^bc^	117.1 ± 1.5 ^b^	76.4 ± 2.9 ^c^	120.0 ± 4.5 ^b^	92.3 ± 4.7 ^a^	70.7 ± 0.9 ^b^
100 °C/15 min	98.2 ± 1.1 ^a^	113.1 ± 2.2 ^b^	89.5 ± 3.8 ^b^	107.4 ± 3.5 ^ab^	91.8 ± 5.3 ^a^	55.2 ± 1.7 ^c^

* SEM: the standard error of the mean. ^a, b, c^ For the same extraction buffer, values in the same column with different letters are significantly different (*p* < 0.05).

## Data Availability

The data presented in this study are available in this article and supplementary materials.

## Supplementary Materials

The following supporting information can be downloaded at: https://www.mdpi.com/article/10.3390/foods11111637/s1, Figure S1: SDS-PAGE analysis of protein fractions from ammonium sulfate precipitation (AMS); Figure S2: Immunoblot of selected fractions during chicken serum albumin (CSA) isolation under reducing (A) and non-reducing (B) conditions.
