# Supplementary material for: Isolation and Characterization of Chicken Serum Albumin (Hen Egg Alpha-Livetin, Gal d 5)
Source: Foods. 2022 Jun 1;11(11):1637. doi: 10.3390/foods11111637 (PMC9180759; doi:10.3390/foods11111637)
Supplement: Supplementary file 1 [file foods-11-01637-s001.zip › foods-1718119-supplementary.pdf]

foods

Supplementary Materials

Isolation and characterization of chicken serum albumin (hen egg alpha-livetin, Gal d 5)

Xingyi Jiang [1], Han Mu [2], Yun-Hwa Peggy Hsieh [1], and Qinchun Rao [1,*]

[1] Department of Nutrition and Integrative Physiology, Florida State University, Tallahassee, Florida, USA. xy1521@outlook.com (X.J.); yhsieh@fsu.edu (Y.-H.P.H.)
[2] Novavax, Inc., Gaithersburg, Maryland, USA. hamu@novavax.com
* Correspondence: Correspondence: qrao@fsu.edu; Tel: 1-850-644-1829.

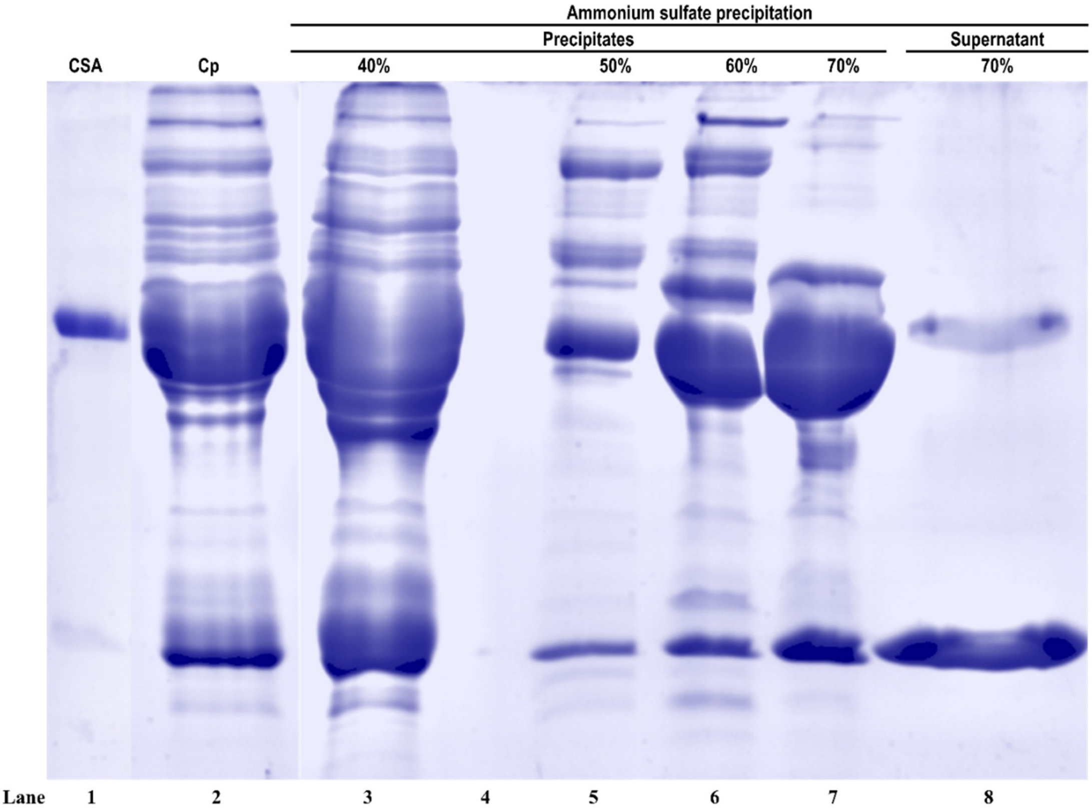

Figure S1. SDS-PAGE analysis of protein fractions from ammonium sulfate precipitation (AMS). The protein loading mass was 1 μg/lane for chicken serum albumin standard (CSA).

The precipitates of different AMS saturation were resuspended in 5 mL of the elution buffer (20 mM Tris-HCl containing 1 M NaCl, pH 9). Chicken blood plasma (Cp) and the products from AMS were 1:1 (mL/mL) mixed with 2× Laemmli buffer containing 5% β-mercaptoethanol. The loading volume was 13 μL/lane.

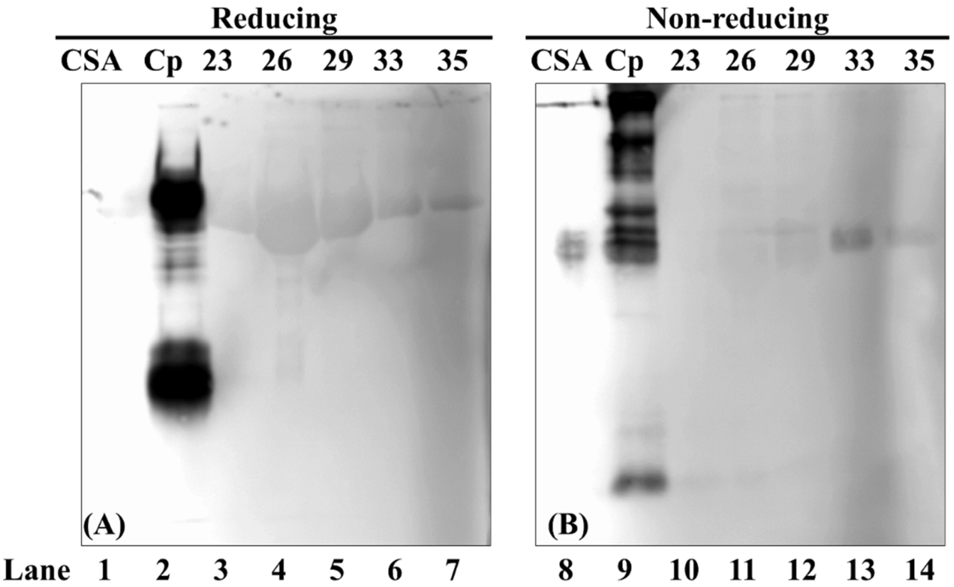

Figure S2. Immunoblot of selected fractions during chicken serum albumin (CSA) isolation under reducing (A) and non-reducing (B) conditions.

The protein loading mass was 1 μg/lane for commercial chicken serum albumin standard (CSA). Chicken blood plasma (Cp) and each fraction from anion exchange chromatography were 1:1 (mL/mL) mixed with 2× Laemmli buffer with or without 5% β-mercaptoethanol. The loading volume was 13 μL/lane. The IgG concentration of goat anti-chicken IgY H & L (alkaline phosphatase, ab6878, RRID: AB_954728, Abcam, Cambridge, UK) was 200 ppm.

Note: The selected fractions from column chromatography were 1:1 (v/v) mixed with 2× Laemmli buffer without or with 5% (v/v) β-mercaptoethanol and separated on SDS-PAGE (4% stacking gel and 12% separating gel). After running at 200 V for 1 h, the proteins on the gel were transferred to a 0.45-μm nitrocellulose membrane (Bio-Rad Laboratories, Hercules, CA). After blocking the membrane using BSA-PBST, the membrane was further incubated with 200 ppm of goat anti-chicken IgY H & L (alkaline phosphatase, ab6878, RRID: AB_954728, Abcam, Cambridge, UK). Color development was performed using the Bio-Rad Immuno-Blot AP Colorimetric kit.
